# Supplementary material for: Early Atf4 activity drives airway club and goblet cell differentiation
Source: Life Sci Alliance. 2024 Jan 4;7(3):e202302284. doi: 10.26508/lsa.202302284 (PMC10766780; doi:10.26508/lsa.202302284)
Supplement: Supplementary file 2 [file LSA-2023-02284_TableS1.docx]

**Supplementary Table 1. Atf4 shRNA oligonucleotides sequence.**

| **shRNA** |  | **Sequence** |
| --- | --- | --- |
| **shRNA 1** | F | CCGGCGGACAAAGATACCTTCGAGTCTCGAGACTCGAAGGTATCTTTGTCCGTTTTTG |
|  | R | AATTCAAAAACGGACAAAGATACCTTCGAGTCTCGAGACTCGAAGGTATCTTTGTCCG |
| **shRNA 2** | F | CCGGCTAGGTCTCTTAGATGACTATCTCGAGATAGTCATCTAAGAGACCTAGTTTTTG |
|  | R | AATTCAAAAACTAGGTCTCTTAGATGACTATCTCGAGATAGTCATCTAAGAGACCTAG |
| **shRNA 3** | F | CCGGCCAGAGCATTCCTTTAGTTTACTCGAGTAAACTAAAGGAATGCTCTGGTTTTTG |
|  | R | AATTCAAAAACCAGAGCATTCCTTTAGTTTACTCGAGTAAACTAAAGGAATGCTCTGG |
