# Supplementary material for: Early Atf4 activity drives airway club and goblet cell differentiation
Source: Life Sci Alliance. 2024 Jan 4;7(3):e202302284. doi: 10.26508/lsa.202302284 (PMC10766780; doi:10.26508/lsa.202302284)
Supplement: Supplementary file 3 [file LSA-2023-02284_TableS2.docx]

| **Name** |  | **Sequence** | **Name** |  | **Sequence** |
| --- | --- | --- | --- | --- | --- |
| **Trp63** | F | CAGATTCAGAACGGCTCCTC | **Igf1** | F | CTCTTCTACCTGGCGCTCTG |
|  | R | GATGGAGAGAGGGCATCAAA |  | R | AAGTAAAAGCCCCTCGGTCC |
| **Krt5** | F | CATCGATTGCACCTGCTCTA | **Ptger4** | F | GATCGAACCGTGAGCTCCAA |
|  | R | TCCAGCAGCTTCCTGTAGG |  | R | CACCACCCCGAAGATGAACA |
| **Foxj1** | F | GTTTCATCCACCCATGTTCC | **Runx2** | F | CATGGTGGAGATCATCGCGG |
|  | R | GTAGGACCCTTCTGGGCTTC |  | R | ACGGGCAGGGTCTTGTTG |
| **Mcidas** | F | CAGATCAAGGCAACAACGAA | **Nell1** | F | AGATCCACTCAGCCCCTCAT |
|  | R | TTAGGGTCACGATTGTGCAG |  | R | AGCTCCCGGATCGACAGTAT |
| **Scgb1a1** | F | CATCATGAAGCTCACGGAGA | **Smad5** | F | ACCTGAGCCACAATGAACCG |
|  | R | AGGTGAGATGCTCGCAGTTT |  | R | AGCAGGGGAAGGAGGATAGG |
| **Scgb3a2** | F | TTCCCTCATTTGATCCCTTG | **Bmpr1b** | F | AAGTGGCGTGGAGAAAAGGT |
|  | R | CACGTAGCAAAGGCTTCTCC |  | R | TTCTCATGCCGCATCAGGAC |
| **Spdef** | F | CAGGGCCTGTCTGCTTTCTA | **Irf7** | F | CCGGTGATCTTTCCCAGTCC |
|  | R | CATCGATTGCACCTGCTCTA |  | R | CCTCCCAGTACACCTTGCAC |
| **Muc5ac** | F | ACACAACCTCCTCAGTCCCT | **Oas1** | F | ACTCAAGGGCAAGTCAGACG |
|  | R | GTGGTAGAACTGACTGGGGC |  | R | CTTGAAGCTCAGAGACCGGG |
| **Irf1** | F | CCTGCCAGACATCGAGGAAG | **Oas2** | F | TGCTCACTGTGTATGCCTGG |
|  | R | TTTCCTCTGGTTCCTGGTGAG |  | R | TGTTCCGGACTGTCTCATCC |
| **Irf2** | F | AATTCCCTGCCCGACATTGA | **Oas3** | F | GGGATCTTGGATTTGGGCCA |
|  | R | AAGGTCGTTCGGATAAGGGC |  | R | GGATCTTGGTGGCTGCTTCT |
| **Irf3** | F | AATGGGAGTTCGAGGTGACC | **Mx2** | F | GTATGAGGAGAAGGTGCGGC |
|  | R | GTCCGTCAGAAACCCCTCAG |  | R | TCCCCAATGACAGCGATGG |
| **Elf3** | F | GCTGTACTCCACCTTGCAGA | **Ifit1** | F | TGAAGCCTTGATCCAGAGCG |
|  | R | GGAGAAGTCGATGGAGCTGG |  | R | TCCTTGCACACCTTCTCCAC |
| **Egr1** | F | CTTACCCGCCATATCCGCAT | **Ifit2** | F | AGTCCTCTTGGCACTGAAGC |
|  | R | GGATGTGGGTGGTAAGGTGG |  | R | GCAATAAAACCTGGCTGCCC |
| **Epas1** | F | CTATGGACGGCGAGGACTTC | **Ifit3** | F | AAGCCAAAGGACCCAGAGTG |
|  | R | CCAACTGCTGCGGGTACTTA |  | R | ATCCTCAGCAGTTTCAGGGC |

**Supplementary Table 2. qPCR oligonucleotides sequence.**
